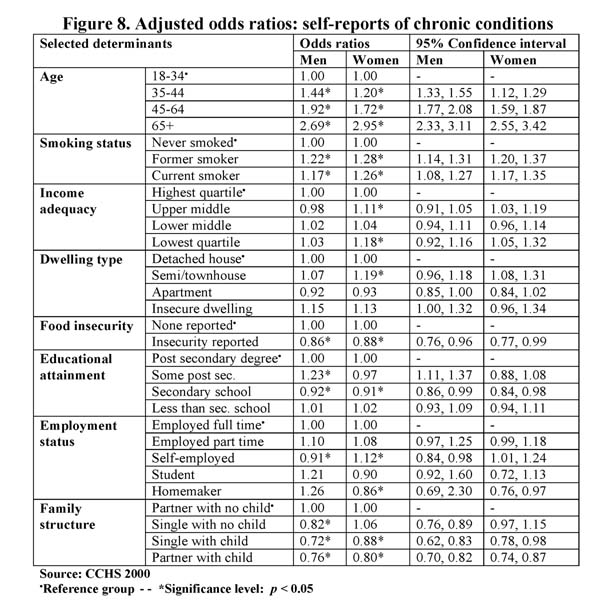# Integrating Socio-Economic Determinants of Canadian Women's Health

**DOI:** 10.1186/1472-6874-4-S1-S34

**Published:** 2004-08-25

**Authors:** Bilkis Vissandjee, Marie Desmeules, Zheynuan Cao, Shelly Abdool

**Affiliations:** 1 School of Nursing Sciences, University of Montreal, Montreal, Canada; 2 Centre for Chronic Disease Prevention and Control, Health Canada, 120 Colonnade Rd, Ottawa, Canada; 3 Centre for Chronic Disease Prevention and Control, Health Canada, 120 Colonnade Rd, Ottawa, Canada; 4 School of Nursing Sciences, University of Montreal, Montreal, Canada

## Abstract

**Health Issue:**

The association between a number of socio-economic determinants and health has been amply demonstrated in Canada and elsewhere. Over the past decades, women's increased labour force participation and changing family structure, among other changes in the socio-economic environment, have altered social roles considerably and lead one to expect that the pattern of disparities in health among women and men will also have changed. Using data from the CCHS (2000), this chapter investigates the association between selected socio-economic determinants of health and two specific self-reported outcomes among women and men: (a) self-perceived health and (b) self-reports of chronic conditions.

**Key Findings:**

The descriptive picture demonstrated by this CCHS dataset is that 10% of men aged 65 and over report low income, versus 23% of women within the same age bracket. The results of the logistic regression models calculated for women and men on two outcome variables suggest that the selected socio-economic determinants used in this analysis are important for women and for men in a differential manner. These results while supporting other results illustrate the need to refine social and economic characteristics used in surveys such as the CCHS so that they would become more accurate predictors of health status given that there are personal, cultural and environmental dimensions to take into account.

**Recommendations:**

Because it was shown that socio economic determinants of health are context sensitive and evolve over time, studies should be designed to examine the complex temporal interactions between a variety of social and biological determinants of health from a life course perspective. Examples are provided in the chapter.

## Background

The association between socio-economic determinants and health has been amply demonstrated in Canada and elsewhere,[1-7], [8,9] the socio-economically better off generally performing better on most measures of health status, including self-reports of health. Studies examining differences between women and men in the relation between socio-economic status and mortality/morbidity have directed attention to socio-economic gradients in health as one potential explanation for gender differences in health. In addition, over the past few decades women's increased labour force participation, their access to higher education, and an evolving family structure have altered social roles considerably. Therefore, the pattern of disparities in health among women and men may have also been modified in concert with these social changes.[6,24-26] This is well outlined in the chapter of this Report that discusses the social context of women's health[75].

In addition to Walters,[7] Macintyre and Hunt[4] point out that in the field of socio-economic inequalities in health, there is a need to examine the specific nature of gender biases, including the way in which social classifications were developed for women and for men (e.g. assessing whether marital and parental roles were accounted for in a gender-sensitive manner).[14,27-32] Taking a variety of socio-economic determinants into account, a number of chapters in this Report demonstrate the relation between sex and gender and health outcomes, ranging from personal health practices (e.g. smoking behaviour) to depression, cardiovascular disease, fertility, medication use and mortality. These results present additional evidence for the existence of socio-economic gradients among women and men in a range of health measures and raise additional questions regarding the specific patterns of these socio-economic gradients in health.

Extensive discussions of potential advantages and disadvantages of the measurement of socio-economic position in relation to health have been presented in excellent reviews by Liberatos et al.,[42] Krieger et al.,[13] Lynch and Kaplan[8] and Berkman and MacIntyre.[43] A number of other studies in Britain, Finland, the United States and Canada have further explored the differential contribution of socio-economic determinants in explaining differences among women and men.[7,44-48] A key issue is whether the association between one socio-economic determinant (e.g. income) and health is entirely or partly explained by other socio-economic determinants (e.g. employment status and education). For instance, it is known that education, occupation and employment status influence both income and health, but the direction of the relation between income and health is not always clear cut. [32-34] Both a causal and reverse relation are possible in this case, which points to the need for improved methods of data collection and analyses to address this issue. [35-38] A number of investigators have demonstrated the ill effects on health of poverty and other adverse conditions such as income inequality, unemployment, job insecurity, lack of social support, social discrimination/exclusion and harmful personal health practices. [18-23]

With respect to the international perspective, Moss[49] highlights the priority of examining socio-economic determinants of women's and men's health. She argues that in the decades between 1973 and 1993, there have been periods of striking growth in developed nations paralleled by increasing socio-economic disparities in health. It has been postulated that societies with high levels of inequality in the distribution of income also have larger health status differences associated with income, measured at either the individual or household level. [31-34] Even after adjustment for several socio-economic determinants such as employment status, education, social class and housing,[36,37,39-41] an inverse relation has been reported between income and 1) health, [34-36] 2) mortality[37,38] (see chapter on mortality in this Report[76]), 3) morbidity[33,40,46] and 4) self-perceived health status.[41,44,50,51]

Krieger et al.[3,13] as well as Macintyre and Hunt[4,14] employ the term "socio-economic position" to refer to the social and economic determinants that influence what position(s) women, men and groups hold within the structure of society. These determinants, which are thought to be good indicators of social location of women and men, were shown to have an influence on health and its perceptions by a specific individual.[8,10-13] Women and men may be 1) differentially exposed to a variety of health risks, 2) differentially vulnerable to various features of the physical and social environment and 3) have differential access to various resources and support systems. Therefore, it is important to gain a better understanding of gender-sensitive pathways (biological and social) associated with specific health outcomes.

Other mechanisms by which gender inequalities in health can occur have been outlined by Diderichsen.[73] His framework delineates four main mechanisms – social stratification, differential exposure, differential susceptibility and differential consequences. As well as these mechanisms, Frank suggests the consideration of a more distal mechanism by which health inequality occurs in a diverse group – that is, the differential "cultures" of symptom expression/reporting, as, for example, may well be the case for women and men's tendencies to report outcomes such as health, pain and some chronic diseases.[74] Lastly, in order to advance our understanding of this area, it is vitally important to develop an optimized set of gender-sensitive indicators that can be practically applied to study such questions.[3,14-17]

Perceived health incorporates a variety of physical, cultural and emotional components of health, which, taken together, comprise individual "healthiness".[49] As a broad indicator of health-related well-being, self-assessment of health has been extensively used within epidemiological and sociological studies and is recognized as being a robust measure of health status.[16,17,33,44] A simple question is often used to assess the respondent's own health as being "excellent, very good, good, fair or poor, compared to other persons their own age"[51-56]. Although such a subjective measure cannot precisely reflect every dimension of health in every population and may not be as reliable as some other more complex indices, it is nonetheless considered an adequate and powerful measure of global health.[16,50-52] Studies based on surveys that assessed self-reported health and chronic conditions, as well as results from a number of chapters included in this Report, show that health perception varies among women and men according to a number of individual characteristics. [53-56] Moreover, a WHO report has recently recommended the use of self-perceived health measures for comparative purposes between women and men.[56]

Accordingly, the present chapter investigates the association between selected socio-economic determinants of health and two specific self-reported outcomes among women and men: (a) self-perceived health and (b) self-reports of chronic conditions. These new analyses provide gender-relevant and gender-sensitive information, not previously reported, on the relation between socio-economic status and health among women and men.

## Methods

### Data Sources and Measures

Cross-sectional data from the Canadian Community Health Survey (CCHS) – Cycle 1.1 (2000) were analyzed for the purposes of this chapter. The CCHS is a repeated, cross-sectional household-level survey that effectively replaces the cross-sectional component of the National Population Health Survey (NPHS). The total sample comprised 125,574 individuals aged 12 years and older at the time of data collection in 2000–2001. The sample used for the purpose of this chapter included women and men aged 18 to 65 years old and over. Accounting for the complex sample design and using the CCHS integrated weighting strategies,[58] it is expected that the final results could be generalized to the entire Canadian population.

### Dependent Variables

1. Self-rated health: respondents assessed their health as excellent, very good, good, fair or poor; in order to assess the profile of the more vulnerable populations, excellent, very good and good health ratings were combined into one group and fair and poor health ratings into another, which is referred to hereafter as poor-rated health;

2. Self-reported chronic conditions were assessed on a dichotomous scale as 1 or more, or no chronic condition (yes, no)* (*The list of the reported chronic conditions can be found in Appendix 1 at the end of this chapter).

### Independent Variables

1. *Age *– four continuous age groups were selected to reflect different periods across the lifespan and workforce participation: 18–34, 35–44, 45–64, 65+;

2. *Family structure *– four categories: single without child, partner without child, single with child, and partner with child;

3. *Educational attainment *– four categories of the highest level of education attained by the respondent: less than secondary school, secondary school, some post secondary school and post-secondary school;

4. *Income adequacy *– four categories: lowest income quartile, lower middle income quartile, upper middle-income quartile, highest income quartile;

5. *Food insecurity *– a dichotomous variable asking whether the respondent had had some food insecurity in the previous 12 months;

6. *Dwelling type *– four categories of the current type of dwelling of the respondent at the time of the survey: detached house, semi-detached/townhouse, apartment and self-reported insecure dwelling; the latter variable included self-reports of living in an institution, a mobile home, or some form of collective dwelling;

7. *Employment status *– a categorical variable based on the respondent's main activity in the previous 12 months and comprising five categories: self-employed (full and part-time), employed full-time, employed part-time, student and home-maker as well as a category named "other" for those who did not see themselves as fitting into any of the other categories.

### Statistical Analysis

Frequency procedures were used to create tables and calculate the prevalence estimates for each determinant. In accordance with Statistics Canada's guidelines, estimates that were based on a sample of fewer than 30 were deemed unreliable and were suppressed. Because socio-economic position has been shown to be associated with gender disparities in health, sex-specific logistic regression models were set up for multivariate analysis to evaluate the effects of covariates on the assessment of self-perceived health and the reported presence of chronic conditions. Confidence intervals for weighted estimates were calculated using the bootstrap method.

## Results

**Figure 1:**
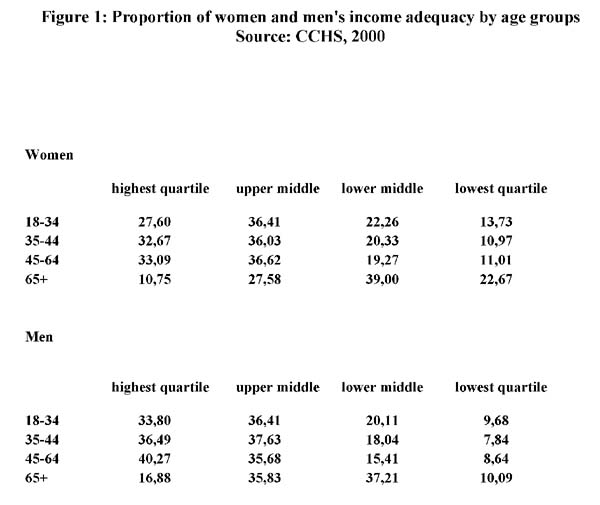


**Figure 2:**
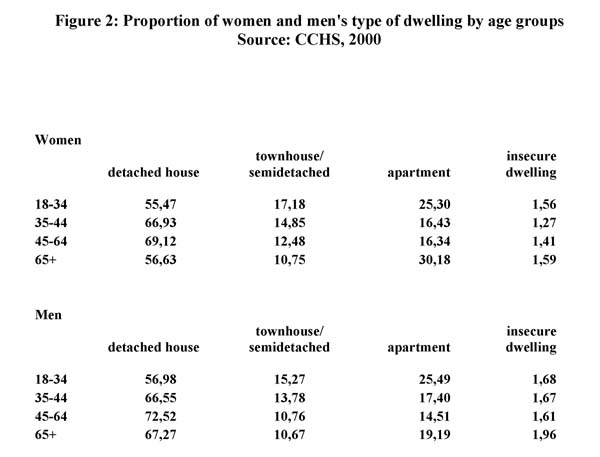


**Figure 3:**
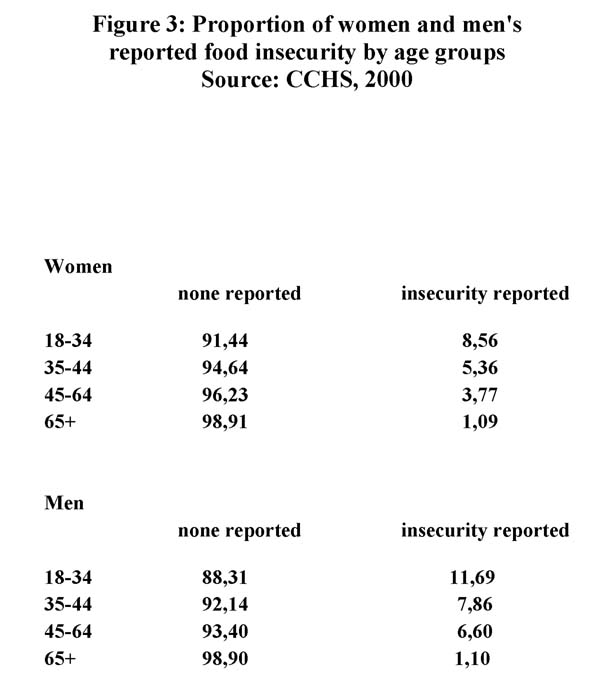


Based on the selected socio-economic determinants of health, the descriptive picture demonstrated by this CCHS dataset is that 10% of men aged 65 and over report low income versus 23% of women within the same age bracket. In addition, 40% of men versus only 33% of women between the ages of 45 and 64 reported high income. While it is not possible to assess whether the respondent owned the dwelling in which he/she lived at the time of the survey, of those aged 65 and over, 67% of men versus 57% of women reported living in a detached house, and 19% of men versus 30% of women reported living in an apartment. There were no differences concerning reports of food insecurity, a large majority of women and men in each age bracket denying any food insecurity within the 12 months preceding the survey (Figures 1, 2 and 3).

**Figure 4:**
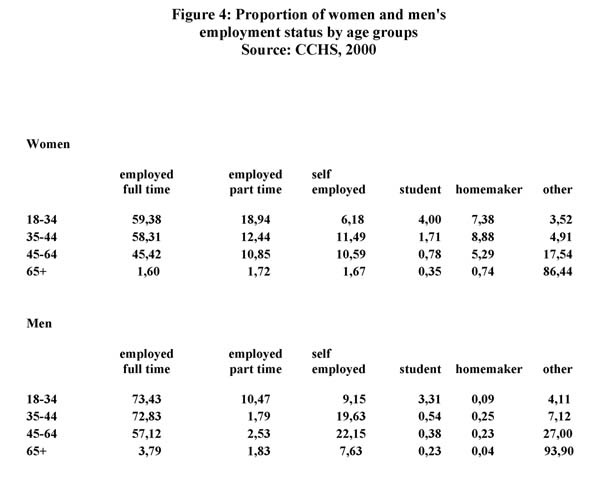


As for employment status, 73% of men in the age bracket 18 to 34 reported full- time employment versus 59% of women within the same age category. As age increased, the proportions of women and men reporting full-time employment decreased in all age groups, but with the overall proportions in full-time employment remaining higher for men. The proportions of men reporting self-employment (full and part-time) were also higher in all age groups (e.g. 22% of men versus 11% of women aged 45 to 64). Conversely, 12% of women aged 35 to 44 and 11% of women aged 45 to 64 reported part-time employment versus 2% and 3% of men respectively in each of the age groups. Being a homemaker also highlighted the differences among women and men (7.0% of women versus 0.1% of men aged 18 to 34; 9.0% of women versus 0.25% of men aged 35 to 44) (Figure 4).

**Figure 5:**
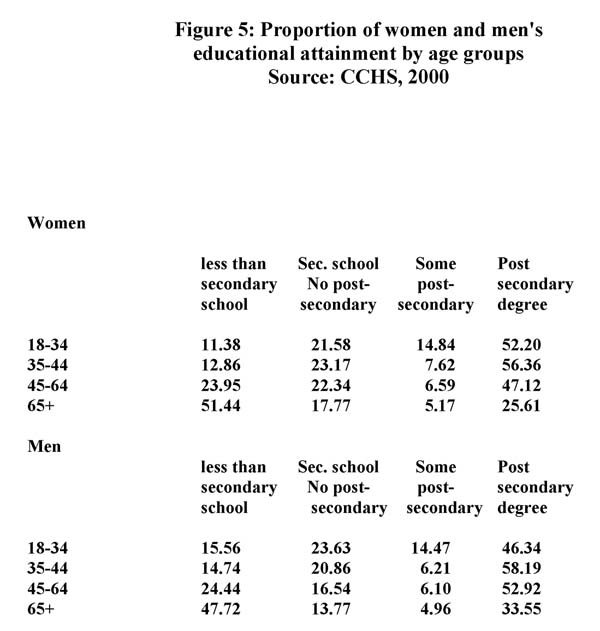


Reflecting the changing nature of women's roles and the increased access to education, the proportions of women and men within various age brackets across different categories of educational attainment were fairly similar. However, 22% of women aged 45 to 64 years reported having attained secondary school versus 17% of men in the same age bracket. On the other hand, the tendency is reversed for the attainment of a postsecondary degree (26% of women age 65 and over versus 34% of men in the same age bracket) (Figure 5). These results may appear surprising because of the relatively high proportion of women and men of this age group reporting attainment of a post-secondary degree. A comparison with the general Canadian population based on Census Canada data (1996) [75] revealed that the CCHS proportions are indeed higher. This may be due to a stronger educational differential in the categories of people participating in the CCHS as opposed to the census. Of note is that if the category "some post-secondary education" is combined with the category "post-secondary degree", the combined proportions from CCHS for both sexes are more comparable with Census Canada regarding attainment of some postsecondary education without the full degree.

**Figure 6:**
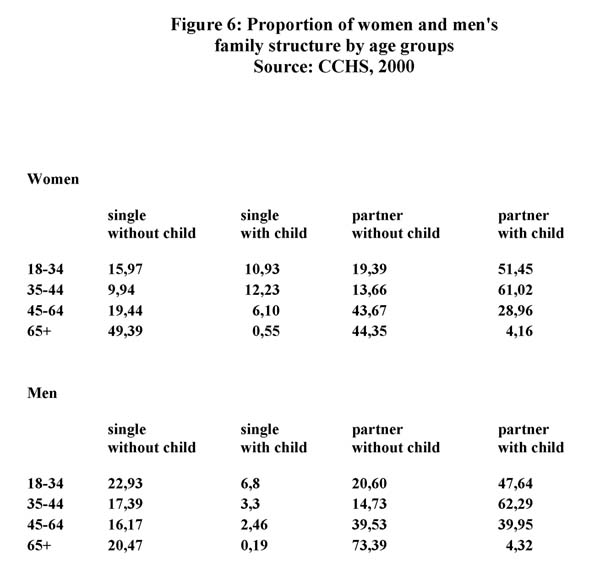


Lastly, in terms of family structure and possibly family support, a greater proportion of women aged 65 and over reported being single (either with or without a child). Among single-headed households in the age group of 35 to 44 years old, 12% of women versus only 3% of men reported living with one or more children (Figure 6).

**Figure 7:**
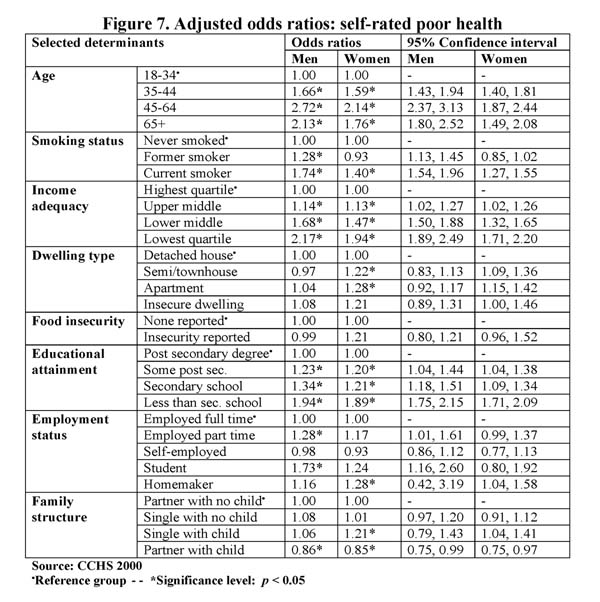


The results of the logistic regression multivariate analyses are presented as odds ratios (OR) together with their 95% confidence intervals (CI) (Figures 7 and 8). Logistic regression analyses are useful for describing the simultaneous relation between a group of continuous and/or categorical independent variables and a dichotomous outcome variable, namely self-rated health and reports of chronic conditions.[68] The relative odds expresses the amount of increase in the outcome that would be produced by one unit increase in the independent variable.[68] In order to avoid obscuring gender differences, as can occur when combining both sexes in multivariate models or age-adjusted health outcomes,[29,36,44,49,50] the models in these analyses were set up separately for women and men. Since a large number of variables were controlled for simultaneously (age and socio-economic position, including employment status, marital status, educational attainment, income adequacy, smoking status, dwelling security and food insecurity[17,35,38,42,43,46,57,60]), it was deemed important to assess the stability of the model in order to rule out biased results due to multicollinearity. Pearson correlations were also performed. Coefficients demonstrated weak to moderate associations between each two given variables, indicating a fairly stable multivariate model.

The analyses, which are adjusted for age, smoking status[60] and the above mentioned socio-economic determinants, demonstrated that women in the lowest income quartile (OR = 1.94, 95% CI: 1.71, 2.20) and reporting lower educational attainment (OR = 1.89, 95% CI: 1.71, 2.09) were significantly more likely to self-rate their health as poor. A gradient by educational attainment is shown for both women and men. Furthermore, there is a consistent pattern of inequality among women, in that the likelihood of reporting poorer health increased as income decreased; a similar pattern was found among men, although the magnitude of the gradient by SES appeared slightly larger among men.

Both women and men who live with a partner and child/children are less likely to report poor health. However, taking into account other material circumstances, women who live in an apartment are more likely to report poor health than those living in a detached house (OR = 1.28, 95% CI: 1.15, 1.42), and women homemakers are more likely to report poor health than women who are employed full-time (OR = 1.28, 95% CI: 1.04, 1.58). In contrast, the type of dwelling did not produce significant differences among men.

In keeping with the pattern of the associations between income and self-rated poor health, women in the lowest income quartile were also more likely to report chronic conditions (OR = 1.18, 95% CI: 1.05,1.32) than those in the highest quartile. Interestingly, women who reported upper-middle income were also more likely to report chronic conditions than those in the highest quartile (OR = 1.11, 95% CI: 1.03, 1.19), and yet no significant associations were shown for lower-middle income women in this regard. In contrast, no significant patterns of association were found between income and reports of chronic conditions for men. These multivariate ORs are smaller than what have usually been found in other studies. This may be because of the simultaneous adjustment of independent variables and also because it is well known that self-report of chronic conditions is frequently underreported by women and men who belong to the lower income groups as a result of underutilization of health services, or increased failure by clinicians to communicate diagnoses in understandable terms or by these patients to remember the diagnoses later.

Educational attainment shows an inconsistent pattern of associations with reported chronic conditions. Women who have completed secondary education are less likely to report chronic conditions than women who hold a postsecondary degree (OR = 0.91, 95% CI: 0.84, 0.98); a similar pattern is observed for men. Unlike self-rated health, being a homemaker does not make a woman more likely to report chronic conditions; however, women who are self-employed (OR = 1.12, 95% CI: 1.01, 1.24) are more likely to report chronic conditions than women who are employed full-time. As for men, those who are self-employed are less likely than full-time employed men to report chronic conditions (OR = 0.91, 95% CI: 0.84, 0.98).

Food insecurity is associated with a lower frequency of reported chronic conditions, possibly because of the observed lower prevalence of obesity among respondents reporting food insecurity (data not shown). Other factors such as under reporting or under-detection among participants reporting food insecurities, may also be contributing to the observed association. 

Lastly, both women (OR = 0.80, 95% CI: 0.74, 0.87) and men (OR = 0.76, 95% CI: 0.70, 0.82) who live with a partner and child are less likely to report chronic conditions than those who live with a partner but without a child. Women who are single and live with a child are also less likely to report chronic conditions than those who live with a partner but without a child (OR = 0.88, 95% CI: 0.78, 0.98). In contrast, men who are single, be it with (OR = 0.72, 95% CI: 0.62, 0.83) or without (OR = 0.82, 95% CI: 0.76, 0.89) a child, are less likely to report chronic conditions than those living with a partner but without a child.

## Discussion

Using a large Canadian dataset, we have attempted to gain a better understanding of differences among Canada's women and men in selected socio-economic determinants of health. Despite some limitations acknowledged below, a number of results are consistent with a population health perspective on the social determinants of health [7,19,45,63,67,69] (and see [75]).

One limitation of our analysis is that the use of cross-sectional data makes it difficult to disentangle the direction of causality and thus limits the ability to exclude the potential for reverse causation. [4,6,7] Nonetheless, when determinants such as age, educational attainment, employment status and other material circumstances (e.g. type of dwelling type, food insecurity status) were adjusted for, the association between income and self-rated health remained statistically significant for both women and men, with a gradient observed for those in the lowest income and lower middle income brackets. The shape of the association is mainly linear, with the likelihood of self-rated poor health increasing upon movement down the income ladder. The association between income and self-reports of chronic conditions also remained significant for women after adjusting for the above mentioned factors, whereas this was not the case for men.

The descriptive portrait showed that fewer women than men reported high income and that fewer women were employed full-time. Given major differences in labour market experience as well as the fact that women tend to occupy particular positions in the labour market, [13,23,26,42,43] the lack of a clear gradient in the employment status associations with self-rated health and reports of chronic conditions might reflect gender differences in the measurement of socio-economic position rather than true differences in the association between the two selected health measures and socio-economic position for women and men. Our results, which show variation among women and men in specific employment status categories such as self-employment and being a homemaker, are consistent with results demonstrating that, in general, socio-economic determinants do vary for women and men and hence there may be a different pattern of exposure and experience among women and men. [4,6,8]

Another finding is that when income, employment status and other material circumstances such as dwelling type and assessment of food insecurity were adjusted for, together with age and smoking behaviour, the association between educational attainment and self-rated health remained statistically significant for both women and men.[4,15,18,23,29,32,33] The shape of the association is linear, with the likelihood of self-rated poor health increasing as one moves down the educational attainment ladder. The association between educational attainment and self-reports of chronic conditions was inconsistent, which may be attributable to either measurement artefacts or to differential vulnerabilities among women and men.[4,18,32,33] This is an area that needs to be further explored.[32,36,44]

With respect to family structure, results are mixed. For women, those who live with a partner and a child were less likely than those who lived with a partner and without a child to self-rate their health as poor, but also less likely to report chronic conditions. As for men, being single with or without a child made them less likely to report chronic conditions. These major differences in personal experience, the fact that single, unemployed or part-time employed women are more likely to experience financial difficulties and to report lower educational attainment might reflect gender differences in the measurement of socio-economic position rather than true differences in the relation between the selected health measures and socio-economic position for women and men.[4,6,8,13] With regard to men, these results may be explained by the fact that some men, such as those without families, may be systematically underreporting the presence of chronic conditions. This is likely since, as mentioned earlier, single men are the least frequent users of health services. These results, which need to be interpreted with caution, still confirm the fact that conventional measures of socio-economic position, such as employment status, educational attainment and household income, need to be assessed in a more gender-sensitive manner.

Despite the fact that it would increase the already high number of variables that need to be accounted for, it appears that more refined measures are necessary to account for social determinants such as domestic conditions, working conditions outside the home, access to resources inside and outside the home, access to opportunities or lack thereof, social participation/capital and resilience.

In sum, the results of the logistic regression models calculated for women and men on two outcome variables suggest that the selected socio-economic determinants used in this analysis are important for women and for men in a differential manner. These results, while supporting other findings,[18,19,22,23,31,33,34,38,39,62,63] illustrate the need to refine social and economic characteristics used in surveys such as the CCHS so that they become more accurate predictors of health status, given that there are personal, cultural and environmental dimensions to take into account. A number of authors recognize that social gradients are not limited to income, education and employment status.[18,19,26,35,63,69,70] Research is being carried out focusing on the underlying mechanisms of gender inequalities in the distribution of health by socio-economic position with a view to obtaining a better grasp of these various relational and cross-cutting social determinants.[13,41,47,49,58,59,66,71,72] Researchers concerned with the intersection of gender and socio-economic position highlight the importance of establishing the gender appropriateness of a particular measure and the need for further refinements in these measures to include women and men in the analyses.[22,23,30,36] For example, there are different occupational opportunities for women and men, and conditions of work are as important to women as to men, even though women have been more closely linked to the domestic sphere.[41,47,50]

## Recommendations

Because it was shown that socio-economic determinants of health are context sensitive and evolve over time, it is recommended that studies be developed that would examine the complex temporal interactions between a variety of social and biological determinants of health from a life course perspective, for instance, as follows:

1. the ways in which socio-economic resources are acquired through training and lost through failing health across the life course;

2. the differential in the responsibilities of women and men with respect to the care provided to both the very young and the very old;

3. the consideration of unpaid work and the role of homemaker according to the differential experiences of men and women;

4. the distinction between individual and household income, reflecting women's ability to access this income but be able to make the decision for its use for individual and family health and well-being;

5. the inclusion in family structure of recomposed families and intergenerational households;

6. the need to increase our understanding of the fact that gender-related variability in the way that people "come to know", remember and report outcomes such as chronic conditions and perceptions of one's health is culturally bound;

7. the need to increase our understanding regarding the widely used self-rated health measure and systematic gender differences in the interpretation of the question asked of respondents as well as the influence of potential mediating variables such as depression, a condition known for its gender differences.

In addition to the need to conduct longitudinal studies to assess the differential influence of social and economic determinants on women and men, allowing for temporal changes to be examined, qualitative studies are also of importance in complementing the understanding of the gendered impacts of these complex determinants on women's and men's health.

## Appendix 1: List or self-reported chronic conditions

CCCA_011 = 'HAS FOOD ALLERGIES?'

CCCA_021 = 'HAS ALLERGIES OTHER THAN FOOD ALLERGIES?'

CCCA_031 = 'HAS ASTHMA?'

CCCA_041 = 'HAS FIBROMYALGIA?'

CCCA_051 = 'HAS ARTHRITIS/RHEUMATISM?'

CCCA_05A = 'ATHRITIS/RHEUMATISM: KIND'

CCCA_061 = 'HAS BACK PROB EXCL FIBROMYALGIA/ARTHRITIS?'

CCCA_071 = 'HAS HIGH BLOOD PRESSURE?'

CCCA_081 = 'HAS MIGRAINE HEADACHES?'

CCCA_91A = 'HAS CHRONIC BRONCHITIS?'

CCCA_91B = 'HAS EMPHYSEMA/CHRONIC OBSTRUCT. PULM. DIS.?'

CCCA_101 = 'HAS DIABETES?'

CCCA_111 = 'HAS EPILEPSY?'

CCCA_121 = 'HAS HEART DISEASE?'

CCCA_12J = 'HEART DISEASE: HAS ANGINA?'

CCCA_12K = 'HEART DISEASE: CONGESTIVE HEART FAILURE?'

CCCA_131 = 'HAS CANCER?'

CCCA_141 = 'HAS STOMACH OR INTESTINAL ULCERS?'

CCCA_151 = 'SUFFERS FROM THE EFFECTS OF A STROKE?'

CCCA_161 = 'HAS URINARY INCONTINENCE?'

CCCA_171 = 'HAS A BOWEL DISORDER/CROHNS/COLITIS?'

CCCA_181 = 'HAS ALZHEIMERS DISEASE/OTHER DEMENTIA?'

CCCA_191 = 'HAS CATARACTS?'

CCCA_201 = 'HAS GLAUCOMA?'

CCCA_211 = 'HAS A THYROID CONDITION?'

CCCA_231 = 'HAS PARKINSONS DISEASE?'

CCCA_241 = 'HAS MULTIPLE SCLEROSIS?'

CCCA_251 = 'HAS CHRONIC FATIGUE SYNDROME?'

CCCA_261 = 'SUFFERS FROM MULT. CHEM. SENSITIVITIES?'

CCCA_221 = 'HAS ANY OTHER CHRONIC CONDITION?'

## Figures and Tables

**Figure 8:**